# Relations of Insulin Resistance, Body Weight, Vitamin D Deficiency, SHBG and Androgen Levels in PCOS Patients

**DOI:** 10.3390/biomedicines13081803

**Published:** 2025-07-23

**Authors:** Zsófi Balogh, Szilvia Csehely, Mónika Orosz, Harjit Pal Bhattoa, Zoárd Tibor Krasznai, Tamás Deli, Attila Jakab

**Affiliations:** 1 Faculty of Medicine, University of Debrecen, 4032 Debrecen, Hungary; baloghzsofi0223@gmail.com; 2 Department of Obstetrics and Gynaecology, Faculty of Medicine, University of Debrecen, 4032 Debrecen, Hungary; csehely.szilvia@med.unideb.hu (S.C.); orosz.monika@med.unideb.hu (M.O.); krasznai.zoard@med.unideb.hu (Z.T.K.); deli.tamas@med.unideb.hu (T.D.); 3 Laboratory Medicine Institute, Faculty of Medicine, University of Debrecen, 4032 Debrecen, Hungary; harjit@med.unideb.hu

**Keywords:** PCOS, insulin resistance, vitamin D deficiency, hyperandrogenism, obesity

## Abstract

**Background:** The most common female endocrinopathy is polycystic ovary syndrome (PCOS), affecting 10–20% of women of reproductive age. It is associated with a wide range of hormonal and biochemical abnormalities and long-term metabolic and cardiovascular risks. It is characterized by infertility due to chronic anovulation, hyperandrogenism, polycystic ovarian morphology, and is often associated with insulin resistance (IR) and obesity. Hyperinsulinemia further increases androgen production and reduces sex hormone-binding globulin (SHBG) levels, thereby aggravating symptoms. In addition, vitamin D deficiency is often present in PCOS patients, and increasing evidence suggests that it may also be associated with insulin resistance and hyperandrogenism. **Objective**: This study aimed to evaluate the relationships between insulin resistance, vitamin D deficiency, body mass index (BMI), and androgen levels in women with PCOS. **Method**: A cross-sectional study was conducted in which data from 195 women diagnosed with PCOS and not yet receiving therapy at a gynecologic endocrinology unit of a university-based tertiary clinical center, between 2019 and 2024, were analyzed. The parameters recorded were age, body mass index (BMI), 25(OH) vitamin D levels, androgen hormone levels (testosterone, androstenedione), glucose-insulin responses during a 3-point oral glucose tolerance test (OGTT). Statistical analyses, including linear regression, Pearson, and Spearman correlation tests were used to assess associations between variables. **Results:** The mean age of the patients was 24.8 years (18–42), and the mean BMI was 30.6 kg/m^2^ (17–51). Vitamin D deficiency was observed in 84.1% of patients, hyperandrogenism in 45.8%, and insulin resistance in 44.5%. A significant inverse correlation was found between BMI and vitamin D levels (r = −0.31, *p* =< 0.01) indicating that higher BMI is associated with lower vitamin D status. Similarly, BMI also showed a significant negative correlation with SHBG levels (r = –0.45, *p* < 0.01), suggesting that increasing body weight is linked to reduced SHBG concentrations. In addition, BMI was significantly positively correlated with 2 h insulin levels (r = 0.43, *p* =< 0.01) and with testosterone levels (r = 0.21, *p* = 0.01). These findings suggest that increased adiposity intensifies insulin resistance and is linked to both vitamin D deficiency and elevated androgen levels. Moreover, the combination of hyperinsulinemia and low vitamin D further disrupts hormonal balance by promoting ovarian androgen production and decreasing SHBG levels, thereby increasing the bioavailability of testosterone. A significant inverse correlation was found between vitamin D levels and 2 h insulin levels (r = −0.28, *p* =< 0.01), indicating that lower vitamin D status is associated with increased insulin resistance. Furthermore, 2 h insulin levels showed a significant positive correlation with testosterone levels (r = 0.32, *p* =< 0.01), suggesting that greater insulin resistance is linked to higher androgen production. Additionally, vitamin D levels were inversely correlated with testosterone (r = −0.18, *p* = 0.02), demonstrating that a lower vitamin D status may further contribute to the hyperandrogenic environment. Vitamin D levels also showed a significant positive correlation with SHBG concentrations (r = 0.29, *p* < 0.01), indicating that a higher vitamin D status may be associated with increased SHBG levels. In contrast, 2 h insulin levels were inversely correlated with SHBG (r = −0.43, *p* < 0.01), reflecting the suppressive effect of hyperinsulinemia on SHBG production. **Conclusions:** Insulin resistance, BMI, and vitamin D deficiency are closely related to each other and to the severity of PCOS, which is confirmed by the correlations with androgen levels. The revealed relationships draw attention to the special importance of vitamin D supplementation and the correction of carbohydrate metabolism in alleviating the symptoms of the disease and reducing long-term health risks.

## 1. Introduction

Polycystic ovary syndrome (PCOS) is one of the most prevalent endocrine disorders affecting women of reproductive age worldwide, with a prevalence of 10% to 20% depending on the diagnostic criteria applied [1]. The condition is characterized by the presence of at least two of the following features, as defined by the Rotterdam criteria: chronic anovulation (manifested as oligo- or anovulation), clinical or biochemical signs of hyperandrogenism, and polycystic ovarian morphology on ultrasound [2]. Beyond these reproductive manifestations, PCOS is also strongly associated with metabolic complications such as insulin resistance, dyslipidemia, and obesity, which increase the long-term risk of type 2 diabetes mellitus and cardiovascular disease [3,4]. Hyperandrogenism is the defining feature of PCOS and plays a central role in determining the severity of its clinical manifestations. Elevated androgen levels—primarily testosterone, androstenedione, and dehydroepiandrosterone sulphate (DHEAS)—are closely linked to symptoms such as hirsutism, acne, alopecia, and menstrual irregularities [5,6]. The extent of hyperandrogenism reflects the overall endocrine imbalance in PCOS and correlates with both reproductive and metabolic dysfunctions. A key driver of androgen excess is insulin resistance (IR), which is present in a significant proportion of PCOS patients, regardless of their body mass index (BMI) [7]. Hyperinsulinemia, a compensatory response to IR, stimulates ovarian androgen production while simultaneously reducing hepatic synthesis of sex hormone-binding globulin (SHBG), further increasing the bioavailability of free testosterone and worsening symptoms [8,9]. BMI is another crucial factor influencing metabolic and hormonal disturbances in PCOS. Obesity is strongly linked to increased insulin resistance and hyperinsulinemia, which further exacerbate androgen excess [10]. Additionally, a higher BMI is consistently associated with lower levels of 25-hydroxyvitamin D (25-OH-D), as adipose tissue sequesters vitamin D, reducing its bioavailability [11]. Consequently, women with PCOS who have elevated BMI often present with both severe insulin resistance and vitamin D deficiency, creating a metabolic environment that further intensifies hyperandrogenism and its associated clinical symptoms [12]. This relationship highlights the importance of BMI not just as an indicator of obesity, but as a key modulator of endocrine and metabolic dysfunction in PCOS. Vitamin D deficiency has gained increasing attention in PCOS research, as evidence suggests that it may contribute to insulin resistance and ovarian dysfunction [13,14,15]. The most reliable indicator of vitamin D status, 25-OH-D, reflects both dietary intake and skin synthesis, making it a valuable biomarker in clinical studies [16]. Several reports have shown a high prevalence of vitamin D deficiency among women with PCOS, and emerging studies suggest that low vitamin D levels may worsen insulin resistance and disrupt ovarian function, potentially influencing folliculogenesis, steroidogenesis, and immune regulation within the ovary [3,5].

While PCOS is a multifactorial endocrine disorder, most studies have examined isolated associations, such as BMI and insulin resistance, or vitamin D deficiency and metabolic dysfunction. Few have investigated how obesity, insulin levels, and vitamin D status interact to influence hyperandrogenism, the main driver of symptom severity. This study addresses that gap by offering an integrated analysis of these factors. Emerging evidence also suggests that vitamin D may play a dual role in modulating both insulin sensitivity and ovarian steroidogenesis. We propose a synergistic model wherein obesity exacerbates insulin resistance and lowers vitamin D bioavailability; insulin resistance promotes androgen excess; and vitamin D deficiency contributes to both insulin dysfunction and androgen production.

Therefore, the purpose of this study is to investigate the relationships between insulin resistance, vitamin D deficiency, BMI, and hyperandrogenism (assessed using both serum testosterone and SHBG levels) in PCOS patients. By analyzing clinical, biochemical, and metabolic data from women diagnosed between 2019 and 2024, we aim to clarify the extent to which insulin levels and vitamin D status contribute to androgen excess. Ultimately, our findings may enhance diagnostic precision and support the development of targeted therapeutic interventions that address both the reproductive and metabolic complications of PCOS.

## 2. Materials and Methods

### 2.1. Study Design and Participants

A cross-sectional study was conducted at our university-based tertiary clinical center’s gynecologic endocrinology unit (Department of Obstetrics and Gynecology). The study included 195 women diagnosed with PCOS between 2019 and 2024 based on the Rotterdam criteria [2]. Patients who had received prior treatment for PCOS (Metformin, Myo-inositol, Vitamin D supplementation, or any form of hormonal contraception) were excluded. Furthermore, patients were excluded if laboratory test results confirmed the presence of congenital adrenal hyperplasia, hypercortisolism, hypothyroidism, or hyperprolactinemia.

### 2.2. Data Collection

Data on age, BMI, 25(OH) vitamin D [25(OH)D] levels, androgen hormones (testosterone, androstenedione, DHEA), SHBG, FSH, LH, E2, PRL, TSH, and glucose-insulin levels from a 3-point oral glucose tolerance test (OGTT) were collected. A 75 g oral glucose tolerance test (OGTT) was performed following an overnight fast. Venous blood was collected at 0, 60, and 120 min for glucose and insulin measurement. Insulin resistance was estimated using the 2 h insulin levels. In the absence of a universally accepted diagnostic consensus, we followed the guideline suggesting that a 120 min post-load insulin level above 80 μU/mL indicates insulin resistance, and values exceeding 300 μU/mL reflect severe insulin resistance [17]. Hyperandrogenism was defined as elevated serum levels of at least one of the following androgens: total testosterone (>2.9 nmol/L), androstenedione (>3.4 μg/L), or dehydroepiandrosterone sulfate (DHEA-S) (>9.3 μmol/L). In women with regular cycles, sampling was performed on menstrual cycle days 2–5, in line with standard clinical protocols for hormonal evaluation. Hormone levels were measured using validated laboratory methods (electrochemiluminescence immunoassay or chemiluminescent immunoassay). Serum estradiol, testosterone, sex hormone-binding globulin, DHEA-S, Prolactin, FSH, LH, and TSH were measured using electrochemiluminescence immunoassay (Roche Diagnostics GmbH, Mannheim, Germany). Serum total 25-hydroxyvitamin D, insulin and androstenedione were measured using chemiluminescence immunoassay (DiaSorin Inc., Stillwater, MN, USA). Serum 17-hydroxyprogesterone was measured using the DIAsource 17OH-progesterone ELISA kit (DIAsource ImmunoAssays S.A., Louvain-la Neuve, Belgium). Hypovitaminosis D was defined as 25-OH-D levels < 75 nmol/L as suggested by Dawson-Hughes et al. [18]. All laboratory analyses were performed using standardized methods.

### 2.3. Statistical Analysis

Linear regression, Pearson (parametric) and Spearman (non-parametric) correlation coefficients were calculated to assess the relationships between vitamin D levels, insulin resistance, and androgen levels. Normal distribution was tested using the Shapiro–Wilk test, the Anderson–Darling test and the Kolmogorov–Smirnov test. Based on these results, Pearson correlation was used for normally distributed variables, while Spearman correlation was applied when variables deviated from normality. To support and validate the correlation findings—particularly in cases where Spearman coefficients suggested weak associations—linear regression analysis was additionally performed. This allowed us to assess the direction and strength of linear relationships while accounting for potential confounding. A *p*-value < 0.05 was considered statistically significant. Given that several variables (such as insulin and SHBG levels) did not follow a normal distribution, descriptive statistics were reported using medians and interquartile ranges (IQR). To visualize these non-normally distributed data, bar charts (column graphs) were created to display the median values, and error bars were used to represent the interquartile range (from the first quartile, Q1, to the third quartile, Q3).

## 3. Results

Among the 195 women included in the study, 72.4% had an elevated BMI (≥25 kg/m^2^), while vitamin D deficiency (defined as 25(OH)D levels below 75 nmol/L) was present in 84.1% of participants. Based on 2 h insulin values, insulin resistance (defined as levels > 80 mU/mL) was identified in 44.5%. Biochemical hyperandrogenism (characterized by elevated serum levels of testosterone (>2.9 nmol/L), androstenedione (>3.4 µg/L), or dehydroepiandrosterone sulfate (DHEA-S; >9.3 µmol/L)) was observed in 44.5% of patients. The following sections present detailed analyses of the associations between these parameters, focusing on the relationships between vitamin D status, insulin levels, BMI, and androgen excess.

### 3.1. Characteristics of PCOS Patients Based on BMI

Table 1 presents a comprehensive overview of the key metabolic and hormonal parameters of the study population, stratified by BMI categories: underweight (BMI < 18.5 kg/m^2^), normal weight (BMI 18.5–24.9 kg/m^2^), overweight (BMI 25.0–29.9 kg/m^2^), mildly obese (BMI 30.0–34.9 kg/m^2^), moderately obese (BMI 35.0–39.9 kg/m^2^), and severely obese (BMI ≥ 40.0 kg/m^2^). The variables assessed include age, average BMI, 2 h insulin levels, HOMA-IR, and 25-hydroxyvitamin D [25(OH)D] concentrations, as well as a range of reproductive and endocrine hormones such as androstenedione, dehydroepiandrosterone sulfate (DHEA-S), total testosterone, sex hormone-binding globulin (SHBG), prolactin, follicle-stimulating hormone (FSH), luteinizing hormone (LH), estradiol (E2), 17-hydroxyprogesterone, and thyroid-stimulating hormone (TSH).

Data are presented as mean ± standard deviation (SD) for variables with a normal distribution and as median with interquartile range (Q1–Q3) for non-normally distributed variables, based on the results of distributional assessment.

### 3.2. BMI Distribution Among PCOS Patients

**Figure 1:**
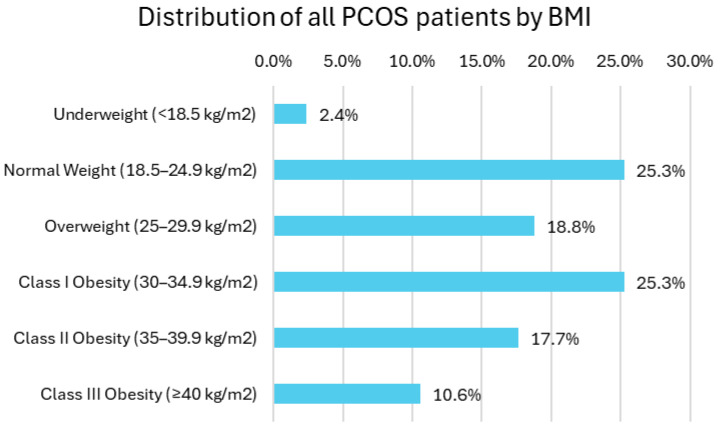
Distribution of all PCOS patients by BMI.

BMI categorization showed the following distribution: 10.6% of patients were classified as severely obese (BMI ≥ 40.0), 17.7% as moderately obese (BMI 35.0–39.9), 25.3% as mildly obese (BMI 30.0–34.9), 18.8% as overweight (BMI 25.0–29.9), 25.3% as normal weight (BMI 18.5–24.9), and 2.4% as slightly underweight (BMI < 18.5) (Figure 1).

### 3.3. Relations of BMI and Vitamin D Level

**Figure 2:**
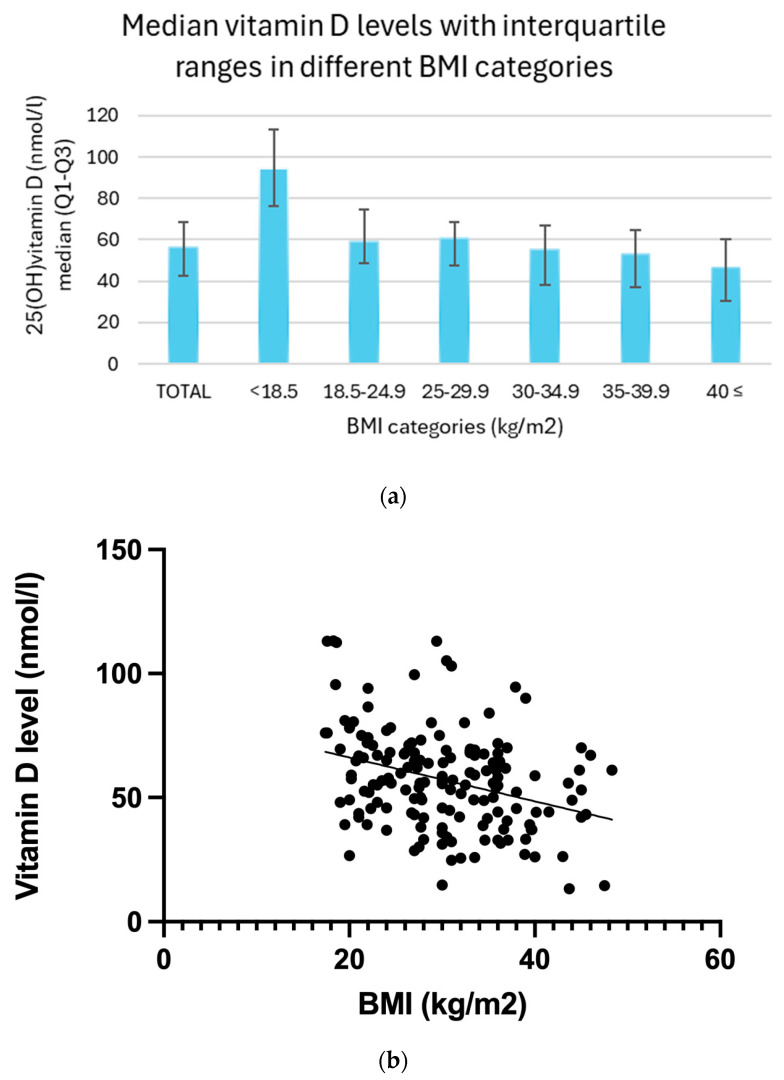
(**a**) Median vitamin D levels with interquartile ranges in different BMI categories. (**b**) Relationship between BMI categories and average vitamin D levels with trendline.

A statistical analysis was conducted to examine the relationship between BMI and vitamin D (25(OH)D) levels. Serum vitamin D concentrations showed a consistent decreasing trend across increasing BMI categories. Underweight participants (BMI < 18.5) exhibited the highest median vitamin D level at 94.5 nmol/L, with an interquartile range (IQR) of 76.0 to 113.2 nmol/L. In the normal weight group (BMI 18.5–24.9), the median concentration was 59.0 nmol/L (IQR: 48.5–74.6 nmol/L), while overweight individuals (BMI 25.0–29.9) had a slightly lower median of 60.9 nmol/L (IQR: 47.7–68.8 nmol/L). Among obese participants, this downward trend became more pronounced: those with Class I obesity (BMI 30.0–34.9) had a median vitamin D level of 55.3 nmol/L (IQR: 38.0–66.8 nmol/L), Class II obesity (BMI 35.0–39.9) showed a median of 53.1 nmol/L (IQR: 37.1–64.4 nmol/L), and individuals with Class III obesity (BMI ≥ 40.0) had the lowest median concentration of 46.6 nmol/L, with an IQR of 30.2 to 60.5 nmol/L. (Figure 2a) Linear regression revealed a significant inverse relationship (*p* < 0.01), indicating that higher BMI values are associated with lower vitamin D levels. Furthermore, a Spearman correlation analysis confirmed this relationship, yielding a moderate negative correlation (r = −0.31, *p* < 0.01) (Figure 2b).

### 3.4. Relation Between BMI and Insulin Resistance

Two-hour insulin levels demonstrated a clear trend across BMI categories, with generally higher median values observed in individuals with increasing body mass. In the underweight group (BMI < 18.5), the median insulin level was 47.1 μU/mL, with an interquartile range (IQR) of 37.7 to 119.5 μU/mL. Among participants with normal weight (BMI 18.5–24.9), the median was 37.2 μU/mL (IQR: 18.0 to 66.0 μU/mL). In the overweight group (BMI 25.0–29.9), insulin levels rose slightly, with a median of 48.4 μU/mL and an IQR of 30.3 to 77.8 μU/mL. A marked elevation was seen in individuals with moderate obesity (BMI 30.0–34.9), whose median insulin level reached 97.7 μU/mL (IQR: 70.0 to 222.7 μU/mL). This trend continued in the severely obese group (BMI 35.0–39.9), with a median of 107.7 μU/mL (IQR: 72.6 to 225.8 μU/mL). Participants with severe obesity (BMI ≥ 40.0) showed a median insulin concentration of 92.7 μU/mL, with a wide IQR of 37.4 to 251.0 μU/mL. Although a slight decline in insulin levels is noted in the severely obese group, this may reflect the limited sample size in these subgroups rather than a true physiological difference.

**Figure 3:**
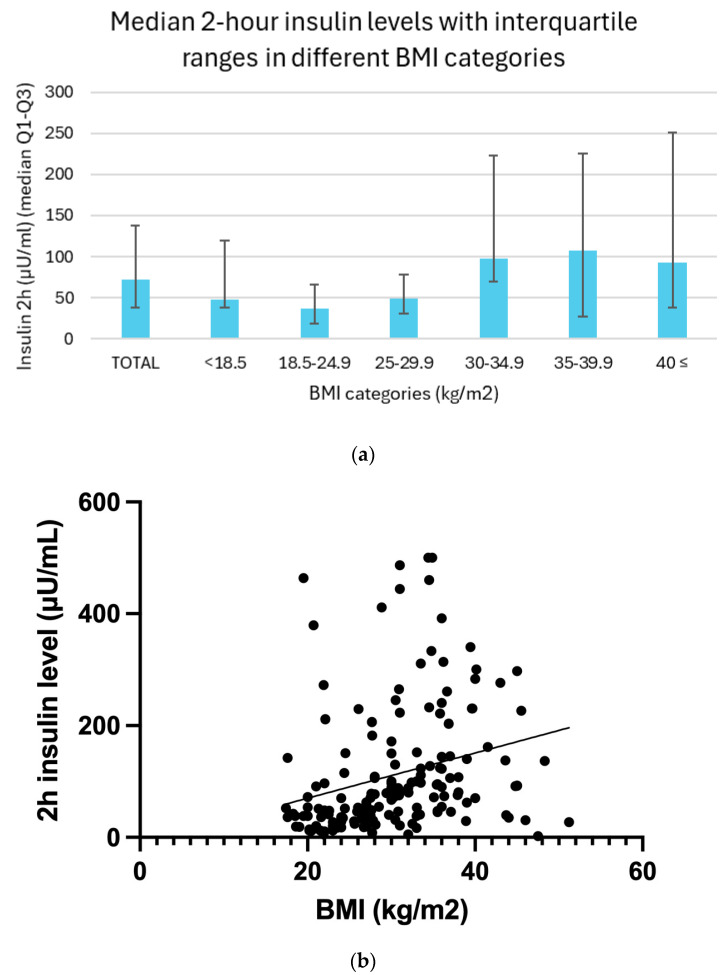
(**a**) Median 2 h insulin levels with interquartile ranges in different BMI categories. (**b**) Relationship between BMI and 2 h insulin levels with trendline.

Between BMI and 2 h insulin levels, a linear regression analysis (*p* = 0.06) and Spearman correlation analysis (r = 0.43, *p* < 0.01) also revealed a moderately significant positive correlation (Figure 3b).

### 3.5. Relation Between BMI and Testosterone Levels

**Figure 4:**
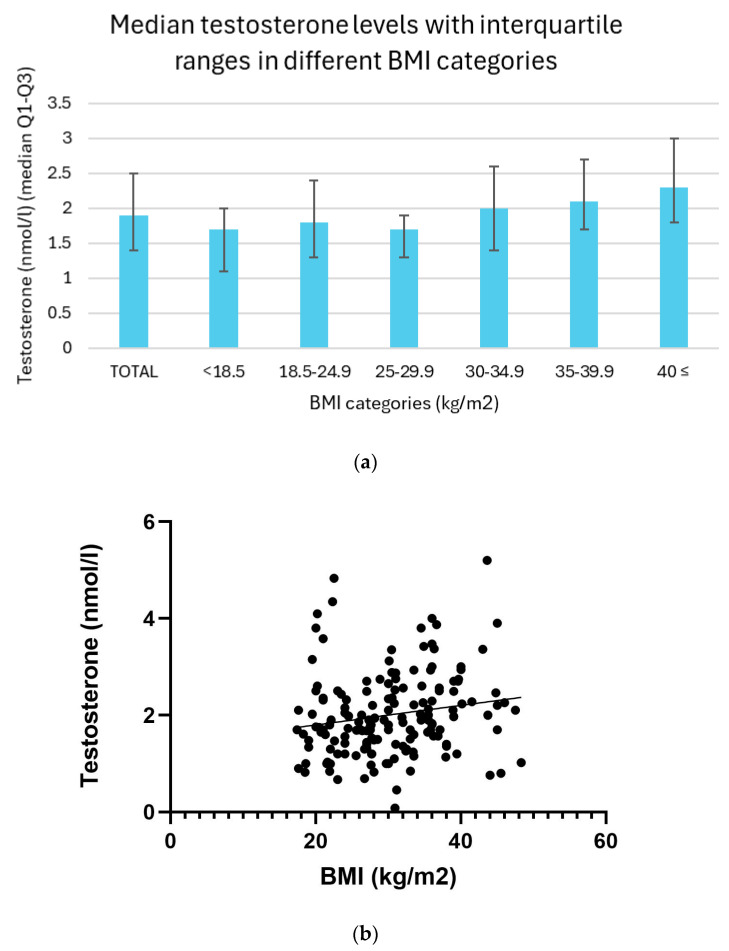
(**a**) Median testosterone levels with interquartile ranges in different BMI categories. (**b**) Relationship between BMI and testosterone levels with trendline.

Serum testosterone levels demonstrated a non-linear pattern across BMI categories. Underweight individuals (BMI < 18.5) exhibited the lowest median testosterone concentration at 1.7 nmol/L, with an interquartile range (IQR) of 1.1 to 2.0 nmol/L. In the normal weight group (BMI 18.5–24.9), the median level was slightly higher at 1.8 nmol/L (IQR: 1.3–2.4 nmol/L), whereas overweight participants (BMI 25.0–29.9) showed a median of 1.7 nmol/L (IQR: 1.3–1.9 nmol/L). A gradual increase in testosterone levels was observed among the obese categories: individuals with Class I obesity (BMI 30.0–34.9) had a median concentration of 2.0 nmol/L (IQR: 1.4–2.6 nmol/L), those with Class II obesity (BMI 35.0–39.9) exhibited a median of 2.1 nmol/L (IQR: 1.7–2.7 nmol/L), and the highest values were found in the Class III obesity group (BMI ≥ 40.0), with a median testosterone level of 2.3 nmol/L (IQR: 1.8–3.0 nmol/L) (Figure 4a).

A linear regression analysis revealed a significant positive correlation between BMI and testosterone levels (*p* = 0.03). Additionally, Spearman correlation analysis revealed a weak but significant positive correlation between them (r = 0.21, *p* =< 0.01) (Figure 4b).

### 3.6. Relation Between BMI and SHBG Levels

**Figure 5:**
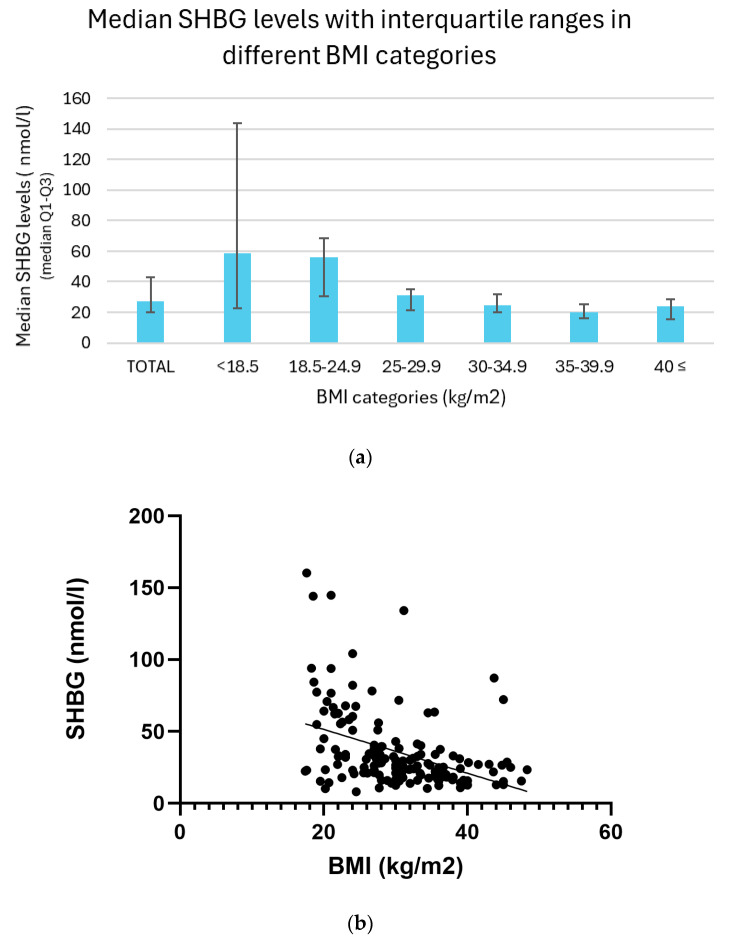
(**a**) Median SHBG levels with interquartile ranges in different BMI categories. (**b**) Relationship between BMI and SHBG levels with trendline.

Serum sex hormone-binding globulin (SHBG) levels exhibited a clear inverse association with increasing BMI categories. The highest median SHBG concentration was observed in underweight individuals (BMI < 18.5), with a value of 58.4 nmol/L and an interquartile range (IQR) of 22.6–143.6 nmol/L. Participants with normal weight (BMI 18.5–24.9) had a slightly lower median of 56.0 nmol/L (IQR: 30.8–68.5 nmol/L). A marked decline was noted among overweight individuals (BMI 25.0–29.9), who presented a median SHBG level of 31.0 nmol/L (IQR: 21.4–35.1 nmol/L). This downward trend continued across the obesity classes: individuals with Class I obesity (BMI 30.0–34.9) had a median of 24.6 nmol/L (IQR: 19.7–31.5 nmol/L), those with Class II obesity (BMI 35.0–39.9) had 19.9 nmol/L (IQR: 16.2–25.1 nmol/L), and those with Class III obesity (BMI ≥ 40.0) showed a median SHBG level of 24.2 nmol/L (IQR: 15.4–28.2 nmol/L). Although the Class III group exhibited a slightly higher median than Class II, this may reflect the limited sample size within this subgroup (Figure 5a).

A significant inverse relationship was observed between BMI and serum SHBG levels. Simple linear regression analysis confirmed this association with a highly significant result (*p* < 0.01). Consistently, Spearman correlation revealed a moderate negative correlation (r = –0.45, *p* < 0.01), indicating that higher BMI values were associated with lower SHBG concentrations across the sample. (Figure 5b).

### 3.7. Vitamin D Level Distribution Among PCOS Patients

**Figure 6:**
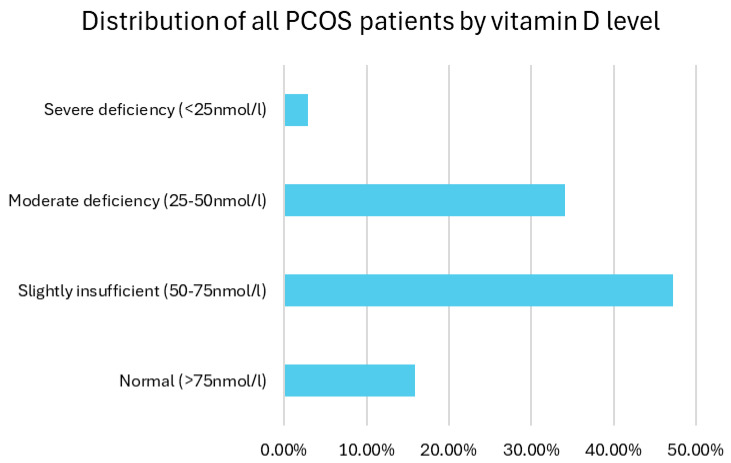
Distribution of all PCOS patients by vitamin D level.

The distribution of vitamin D (25(OH)D) levels in the studied population was as follows: 84.1% of the participants had vitamin D levels below 75 nmol/L, indicating insufficiency or deficiency. Specifically, 2.8% of participants had vitamin D levels below 25 nmol/L (indicative of severe deficiency), 34.1% had levels between 25 and 50 nmol/L (moderate deficiency) and 47.2% had levels between 50 and 75 nmol/L (slightly below normal). Only 15.9% of participants had normal vitamin D levels above 75 nmol/L. A total of 15.9% had levels above 75 nmol/L (considered normal) (Figure 6).

### 3.8. Two-Hour Insulin Level Distribution Among PCOS Patients

**Figure 7:**
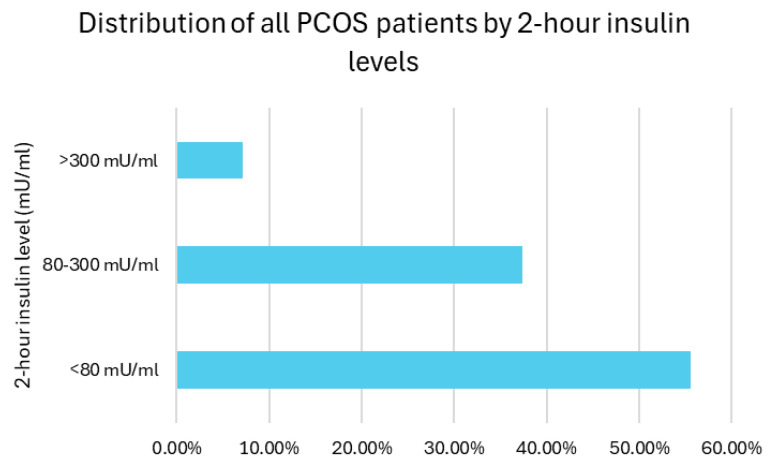
Distribution by 2 h insulin levels.

As no diagnostic consensus was available to detect IR, the distribution of 2 h insulin levels among the patients was categorized as suggested in the study period: 55.5% of participants had insulin levels below 80 mU/mL, 37.4% had levels between 80 and 300 mU/mL (moderate IR), and 7.1% had levels exceeding 300 mU/mL (severe IR). Overall, insulin resistance (defined as 2 h insulin >80 mU/mL) was present in 44.5% of the participants (Figure 7).

### 3.9. Relation Between Vitamin D Levels and 2 h Insulin Levels

**Figure 8:**
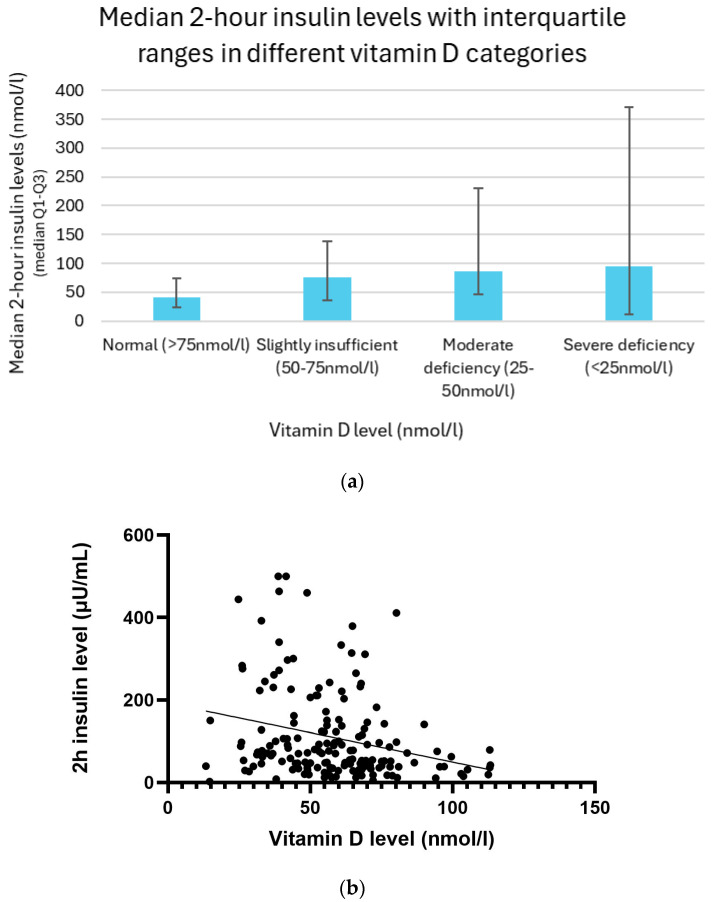
(**a**) Median 2 h insulin levels with interquartile ranges in different vitamin D categories. (**b**) Relationship between vitamin D and insulin levels with trendline.

The two-hour insulin levels increased progressively with the decreasing vitamin D status. Individuals with normal vitamin D levels (>75 nmol/L) had the lowest insulin response, with a median value of 41.1 μU/mL and an interquartile range (IQR) of 23.5–74.6 μU/mL. Among participants with slightly insufficient vitamin D levels (50–75 nmol/L), the median insulin concentration increased to 75.8 μU/mL (IQR: 36.3–137.6). This upward trend continued in the group with moderate deficiency (25–50 nmol/L), where the median reached 85.9 μU/mL (IQR: 46.2–230.4). The highest postprandial insulin values were observed in individuals with severe vitamin D deficiency (<25 nmol/L), showing a median of 94.9 μU/mL and a wide interquartile range of 11.7–370.5 μU/mL (Figure 8a).

The relationship between vitamin D (25(OH)D) and 2 h insulin levels was analyzed using both linear regression and Spearman correlation methods. A linear regression analysis revealed a significant negative relationship between them (*p* =< 0.01). Spearman correlation analysis further supported these findings, revealing a weak but significant negative monotonic relationship (r = −0.28, *p* =< 0.01) (Figure 8b).

### 3.10. Relation Between Vitamin D and Testosterone Levels

**Figure 9:**
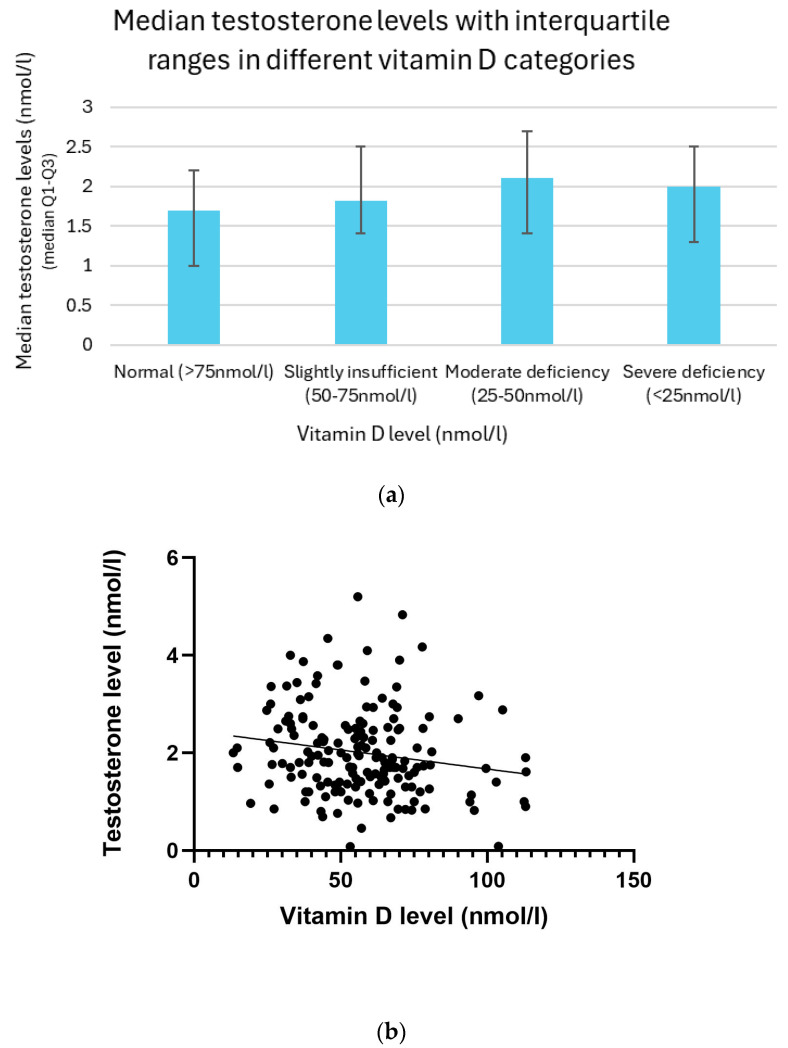
(**a**) Median testosterone levels with interquartile ranges in different vitamin D categories. (**b**) Relationship between vitamin D and testosterone levels with trendline.

Serum testosterone levels exhibited a non-linear distribution across categories of vitamin D status. Participants with sufficient vitamin D levels (>75 nmol/L) showed the lowest median testosterone concentration at 1.7 nmol/L, with an interquartile range (IQR) of 1.0–2.2 nmol/L. Those with slightly insufficient levels (50–75 nmol/L) demonstrated a higher median of 1.8 nmol/L (IQR: 1.4–2.5 nmol/L). The highest median testosterone level was observed in the group with moderate vitamin D deficiency (25–50 nmol/L), measuring 2.1 nmol/L (IQR: 1.4–2.7 nmol/L). Interestingly, in participants with severe deficiency (<25 nmol/L), the testosterone level slightly declined to 2.0 nmol/L (IQR: 1.3–2.5 nmol/L). This apparent decrease in the severely deficient group may be influenced by the smaller sample size or individual variability (Figure 9a).

The association between vitamin D (25(OH)D) levels and testosterone was analyzed using linear regression and Pearson correlation methods. A linear regression analysis revealed a significant negative relationship between vitamin D levels and testosterone (*p* = 0.02). Pearson’s correlation analysis supported these findings, showing a weak but significant negative correlation between them (r = −0.18, *p* = 0.02) (Figure 9b).

### 3.11. Relation Between Vitamin D and SHBG Levels

**Figure 10:**
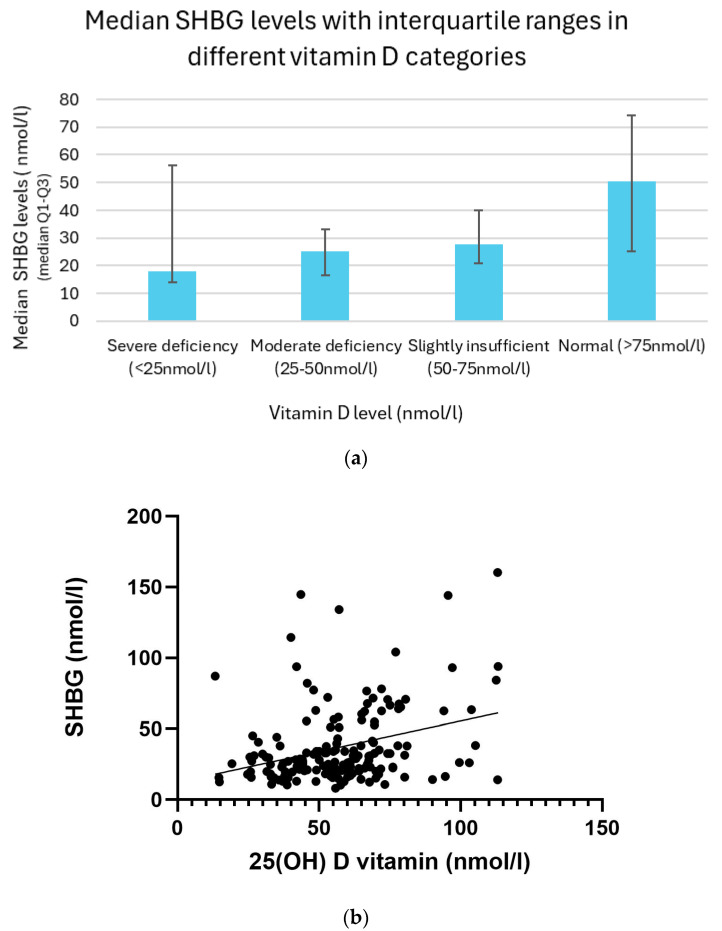
(**a**) Median SHBG levels with interquartile ranges in different vitamin D categories. (**b**) Relationship between vitamin D and SHBG levels with trendline.

Serum SHBG (sex hormone-binding globulin) levels demonstrated an increasing trend in relation to improving vitamin D status. Participants with severe vitamin D deficiency (<25 nmol/L) had the lowest SHBG concentrations, with a median of 18.0 nmol/L and an interquartile range (IQR) of 14.1–56.2 nmol/L. In the group with moderate deficiency (25–50 nmol/L), the median SHBG level rose to 25.2 nmol/L (IQR: 16.5–33.0 nmol/L), while those with slightly insufficient levels (50–75 nmol/L) had a median of 27.9 nmol/L (IQR: 20.8–40.1 nmol/L). The highest SHBG concentrations were observed in participants with sufficient vitamin D levels (>75 nmol/L), with a median of 50.5 nmol/L and IQR of 25.3–74.1 nmol/L (Figure 10a).

A statistically significant positive association was found between vitamin D and SHBG levels. Simple linear regression confirmed the relationship with a highly significant result (*p* < 0.01). In line with this, the Spearman correlation analysis revealed a weak to moderate positive correlation (r = 0.29, *p* = 0.01), further supporting the presence of a consistent and significant trend between the two variables (Figure 10b).

### 3.12. Relation Between 2 h Insulin and Testosterone Levels

**Figure 11:**
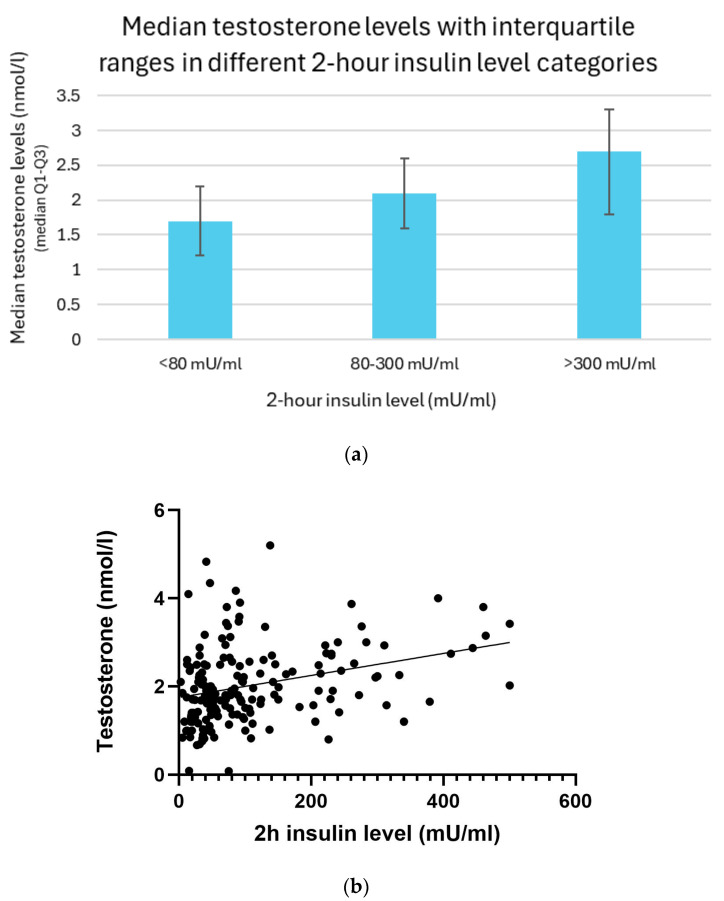
(**a**) Median testosterone levels with interquartile ranges in different 2 h insulin level categories. (**b**) Relationship between 2 h insulin and testosterone levels with trendline.

Testosterone levels increased progressively with higher 2 h insulin concentrations. Participants with post-load insulin levels below 80 mU/mL showed the lowest testosterone concentrations, with a median of 1.7 nmol/L and an interquartile range (IQR) of 1.2–2.2 nmol/L. In individuals whose insulin levels ranged from 80 to 300 mU/mL, the median testosterone level rose to 2.1 nmol/L (IQR: 1.6–2.6 nmol/L). The highest testosterone concentrations were observed in the group with insulin levels exceeding 300 mU/mL, with a median of 2.7 nmol/L and IQR of 1.8–3.3 nmol/L (Figure 11a).

The association between 2 h insulin levels and testosterone was analyzed using the linear regression and Spearman correlation methods. A linear regression analysis revealed a significant positive relationship between them (*p* < 0.01). Spearman’s correlation analysis further supported these findings, showing a moderate positive monotonic relationship (r = 0.32; *p* < 0.01) (Figure 11b).

### 3.13. Relation Between 2 h Insulin and SHBG Levels

**Figure 12:**
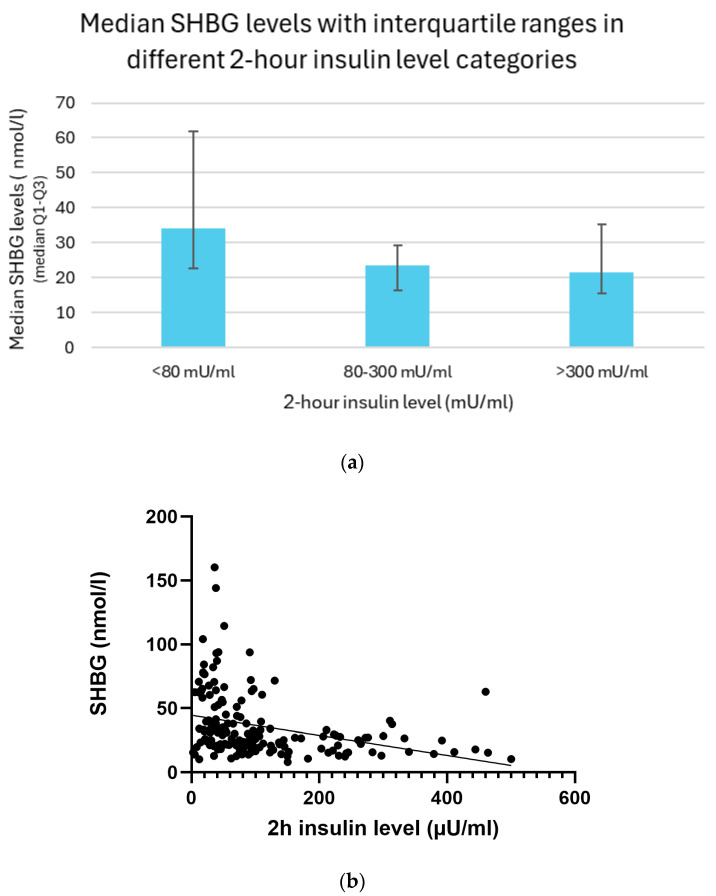
(**a**) Median SHBG levels with interquartile ranges in different 2 h insulin level categories. (**b**) Relationship between 2 h insulin and SHBG levels with trendline.

Serum SHBG (sex hormone-binding globulin) levels were inversely associated with insulin concentrations. Participants with 2 h insulin levels below 80 mU/mL had the highest SHBG concentrations, with a median of 34.0 nmol/L and an interquartile range (IQR) of 22.6–61.8 nmol/L. In contrast, individuals with insulin levels between 80 and 300 mU/mL showed a markedly lower SHBG level, with a median of 23.4 nmol/L (IQR: 16.3–29.2 nmol/L). Those with insulin levels above 300 mU/mL demonstrated a similar, slightly lower SHBG concentration, with a median of 21.5 nmol/L and IQR of 15.5–35.3 nmol/L; this minor difference may be attributed to the relatively small sample size in the highest insulin category (Figure 12a).

A significant inverse relationship was observed between 2 h insulin levels and serum SHBG concentrations. Simple linear regression confirmed this association with a highly significant result (*p* < 0.01). Similarly, Spearman correlation analysis demonstrated a moderate negative correlation (r = −0.43, *p* < 0.01), indicating that higher insulin levels were consistently associated with lower SHBG values. These findings suggest a robust and statistically significant relationship between hyperinsulinemia and decreased SHBG concentrations (Figure 12b).

## 4. Discussion

Our research specifically examined how obesity, elevated insulin levels, and vitamin D deficiency contribute to androgen excess, further exacerbating the clinical manifestations of the syndrome. The findings highlight the significant relationships between these factors in PCOS patients.

Results demonstrate a clear association between obesity and both metabolic disturbances and a less favorable endocrine profile in affected women. In previous research, the Mendelian randomization (MR) analysis provided strong evidence of a causal link between increased BMI and a higher risk of developing PCOS, while also suggesting that the severity of PCOS may, in turn, contribute to higher BMI levels [19]. The observed inverse correlation between BMI and vitamin D levels suggests that increased adiposity is linked to lower vitamin D bioavailability, likely due to sequestration in adipose tissue [20]. This is particularly relevant in PCOS, where both obesity and vitamin D deficiency are prevalent conditions. In 2023, Lejman-Larysz et al. confirmed the significant relationship between these factors and, moreover, they demonstrated that vitamin D not only correlates with BMI but also with metabolic syndrome, waist-to-hip ratio, and waist circumference [21].

Furthermore, our data confirms the well-established association between BMI and insulin resistance. Higher BMI was significantly correlated with increased 2 h insulin levels, reinforcing the role of obesity in exacerbating insulin resistance. Yu et al. (2023) investigated the characteristics and potential mechanisms underlying metabolic disorders in overweight women with PCOS. Their cross-sectional study involved 947 women with PCOS, categorized based on body mass index. The findings revealed that the overweight group exhibited higher insulin resistance indices and a greater prevalence of acanthosis nigricans [22]. This relationship is clinically significant, as insulin resistance is a key driver of androgen excess in PCOS. In 2021, de Medeiros, Rodgers, and Norman found that visceral obesity contributes to adipocyte dysfunction, and dysfunctional adipocyte products (e.g., leptin, adiponectin, chemerin) affect steroidogenic enzyme expression, contributing to hyperandrogenism, insulin resistance, and metabolic issues [23]. Paulukinas, Mesaros, and Penning confirmed the connection between hyperinsulinemia and hyperandrogenism in 2022, when they discovered that the aldo-keto reductase family 1 member C3 (AKR1C3), an insulin driven enzyme, plays a key role in androgen production in adipocytes driven by insulin and identified AKR1C3 as a potential therapeutic target to reduce androgen excess [24]. Hyperinsulinemia promotes ovarian androgen production and simultaneously reduces hepatic SHBG synthesis, leading to higher circulating free testosterone levels. In our study, we found a positive correlation between insulin resistance and hyperandrogenism. Women with higher 2 h insulin levels had significantly elevated testosterone concentrations, underscoring the role of insulin in stimulating ovarian androgen production. Furthermore, a negative correlation was observed between insulin resistance and sex hormone-binding globulin (SHBG) levels. As insulin levels increased, SHBG concentrations decreased, which may contribute to elevated free androgen levels and further exacerbate hyperandrogenic symptoms. Our results, aligning with previous studies, suggest that insulin resistance is not merely a consequence of obesity but an independent contributor to the pathophysiology of PCOS [25,26,27,28]. These results reinforce the concept of a bidirectional relationship between metabolic and hormonal disturbances in PCOS, where insulin resistance and hyperandrogenism mutually exacerbate each other. Given that hyperandrogenism is a key contributor to symptom severity in many PCOS phenotypes, addressing insulin resistance through lifestyle modifications, pharmacological interventions, and, potentially, vitamin D supplementation may support a more effective and individualized management approach.

Vitamin D levels were inversely correlated with both insulin resistance and androgen levels. Patients with lower vitamin D levels exhibited significantly higher 2 h insulin concentrations and increased testosterone levels. This finding supports the hypothesis that vitamin D plays a role in modulating insulin sensitivity and ovarian steroidogenesis. Furthermore, vitamin D levels were positively correlated with SHBG concentrations; as serum vitamin D increased, SHBG levels also rose, suggesting a potential modulatory role of vitamin D in sex hormone regulation. Although the exact mechanisms remain unclear, evidence suggests that vitamin D may play a regulatory role in both glucose metabolism and steroidogenesis, implicating it in the pathophysiology of PCOS. One of the primary ways vitamin D may influence insulin sensitivity is through its action on pancreatic β-cell function and insulin signaling pathways. The active form of vitamin D, 1,25-dihydroxyvitamin D3, binds to vitamin D receptors (VDRs), which are widely expressed in metabolic tissues, including pancreatic islets, adipose tissue, skeletal muscle, and the liver. Through this receptor-mediated action, vitamin D may enhance insulin receptor expression, promote glucose transporter (GLUT-4) translocation, and reduce systemic inflammation—all of which contribute to improved insulin action [29,30]. Krul-Poel et al. (2018) found that low serum 25-hydroxyvitamin D [25(OH)D] levels are significantly linked to higher insulin resistance in women with PCOS—independent of BMI, season, and ethnicity—and also that severe vitamin D deficiency is associated with the lowest levels of HDL cholesterol and apolipoprotein A1 [31]. Li et al. (2021) used the insulinogenic and disposition indices to explore whether vitamin D plays a role in insulin secretion. Their results revealed a significant positive association between serum 25(OH)D levels and the disposition index, a dynamic measure of β-cell function adjusted for insulin sensitivity. This indicates that vitamin D may facilitate early-phase insulin release after glucose stimulation, potentially preventing the compensatory hyperinsulinemia characteristic of PCOS [32]. Beyond its metabolic effects, vitamin D may also exert direct endocrine effects on ovarian function. VDRs are expressed in several components of the reproductive axis, including ovarian theca and granulosa cells, suggesting that vitamin D may influence ovarian steroidogenesis. Behmanesh et al. (2019) reported that vitamin D levels were inversely correlated with serum testosterone, insulin resistance parameters, and body fat mass in women with PCOS. Importantly, supplementation with vitamin D was shown to improve both metabolic and reproductive outcomes, including lipid metabolism and mental well-being, underscoring its systemic role in PCOS management [33]. In 2017, Bakhshalizadeh et al. were the first to demonstrate the unique role of vitamin D3 in modulating steroidogenesis by showing its impact on the regulation of steroidogenic enzyme expression in granulosa cells derived from PCOS patients. They found that vitamin D3 modulated the transcription and activity of key steroidogenesis-related genes, including CYP19A1 (aromatase), StAR (steroidogenic acute regulatory protein), and 3β-HSD, all of which are essential in the biosynthesis and regulation of androgens. These findings support a model in which vitamin D exerts intracellular control over androgen production, potentially mitigating hyperandrogenism at the ovarian level [34]. Moreover, chronic vitamin D deficiency may exacerbate low SHBG levels, thereby increasing the free androgen index and aggravating clinical symptoms such as hirsutism, acne, and menstrual irregularity. It may also contribute to chronic low-grade inflammation and oxidative stress, further worsening insulin resistance and interfering with ovulatory function [35]. The clinical implications of these findings suggest that optimizing vitamin D levels could be beneficial in managing insulin resistance and hyperandrogenism in PCOS patients. According to a recent study, vitamin D3 treatment may be effective either on its own or as an adjunct therapy in managing PCOS. Tóth et al. (2025) showed that vitamin D_3_ supplementation led to improvements in hormonal and metabolic markers (including testosterone, SHBG, and insulin resistance), as well as menstrual cycle regularity in women with PCOS [36].

In summary, our study provides evidence that BMI, insulin resistance, and vitamin D deficiency correlate with the severity of laboratory hyperandrogenism in PCOS. These findings emphasize the importance of a multifaceted approach that includes weight management, insulin-sensitizing therapies, and vitamin D optimization to mitigate the metabolic and endocrine dysfunctions characteristic of the syndrome. From a clinical standpoint, this underscores the need for individualized treatment strategies, such as structured lifestyle interventions (e.g., nutritional counseling, regular physical activity), pharmacological agents like metformin to improve insulin sensitivity, and targeted vitamin D supplementation in deficient patients. Early identification and correction of these modifiable risk factors may not only alleviate hyperandrogenic symptoms such as hirsutism, acne, and menstrual irregularities, but also help prevent long-term complications, including type 2 diabetes and cardiovascular disease. By integrating metabolic, hormonal, and nutritional management, clinicians can adopt a more personalized and effective approach to PCOS care.

### Limitations

This study has several limitations that should be considered. First, its retrospective design inherently limits the ability to infer causality. Data was collected from medical records, which may include inconsistencies or incomplete information. Second, the absence of longitudinal or interventional data restricts our ability to observe temporal changes or effects of treatment interventions. Additionally, confounding factors such as lifestyle, sun exposure, dietary habits, or seasonal variation in vitamin D levels were not controlled for. Future prospective or randomized controlled studies are warranted to validate these findings and explore causal relationships.

## Figures and Tables

**Table 1:** Characteristics of all PCOS patients and subgroups stratified by BMI.

BMI Categories (kg/m^2^)	Total	<18.5	18.5–24.9	25.0–29.9	30.0–34.9	35.0–39.9	≤40.0
Age (yrs) (mean ± SD)	24.8 ± 4.4	24.5 ± 3.6	23.7 ± 3.2	25.1 ± 4.1	25.7 ± 5.4	24.9 ± 3.9	25.2 ± 4.4
Average BMI (kg/m^2^) (mean ± SD)	30.5 ± 8.2	17.7 ± 0.3	21.8 ± 1.6	27.3 ± 1	31.9 ± 1.6	37.0 ± 1.5	44.4 ± 3.0
Insulin 2 h (μU/mL) (median (Q1–Q3))	71.5 (38.0–137.8)	47.1 (37.7–119.5)	37.2 (18.0–66.0)	48.4 (30.3–77.8)	97.7 (70.0–222.7)	107.7 (72.6–225.8)	92.7 (37.4–251.0)
HOMA-IR (median (Q1–Q3))	3.4 (2–5.2)	1.9 (1.2–2.4)	1.7 (1.2–2.6)	2.7 (1.9–3.6)	3.8 (3.0–5.8)	5.2 (4.0–7.5)	5.2 (4.3–8.5)
25(OH) vitamin D (nmol/L) (median (Q1–Q3))	56.4 (42.4–68.7)	94.5 (76.0–113.2)	59.0 (48.5–74.6)	60.9 (47.7–68.8)	55.3 (38.0–66.8)	53.1 (37.1–64.4)	46.6 (30.2–60.5)
Androstenedione (μg/L) (median (Q1–Q3))	3.1(2.4–4.1)	2.9 (2.0–3.7)	2.9 (2.2–4.1)	2.9 (1.8–3.7)	3.4 (2.5–4.2)	3.3 (2.4–4.1)	3.2 (2.7–3.8)
DHEAS (μmol/L) (median (Q1–Q3))	8.8 (6.8–11.5)	7.2 (6.1–11)	9.3 (6.3–11.9)	8.2 (6.9–11.9)	8.3 (6.6–10.6)	9.5 (8.6–12.3)	9.4 (7.6–14.8)
Testosterone (nmol/L) (median (Q1–Q3))	1.9 (1.4–2.5)	1.7 (1.1–2.0)	1.8 (1.3–2.4)	1.7 (1.3–1.9)	2.0 (1.4–2.6)	2.1 (1.7–2.7)	2.3 (1.8–3.0)
SHBG (nmol/L) (median (Q1–Q3))	27.1 (19.7–43.1)	58.4 (22.6–143.6)	56.0 (30.8–68.5)	31.0 (21.4–35.1)	24.6 (19.7–31.5)	19.9 (16.2–25.1)	24.2 (15.4–28.2)
Prolactin (mU/L) (median (Q1–Q3))	15.0 (11.2–21.9)	19.9 (7.9–30.6)	19.0 (13.6–26.7)	15.1 (3.9–6.0)	14.3 (9.8–19.7)	12.8 (11.0–16.5)	14.2 (6.8–18.1)
FSH (IU/L) (median (Q1–Q3))	5.3 (4.5–6.1)	7.0 (6.1–7.8)	5.6 (4.7–6.5)	4.8 (3.9–6.0)	5.0 (3.7–6.1)	5.4 (4.7–6.1)	5.3 (4.4–5.7)
LH (IU/L) (median (Q1–Q3))	10.0 (6.3–15.6)	13.3 (7.7–18.8)	12.0 (7.0–20.3)	7.6 (5.1–14.7)	7.8 (5.0–15.5)	10.8 (6.4–14.4)	8.8 (6.5–12.4)
Estrogen (ng/mL) (median (Q1–Q3))	52.8 (41.3–66.6)	60.6 (51.9–140.3)	52.2 (36.9–70.8)	58.0 (44.2–68.2)	51.0 (40.0–66.9)	50.5 (40.7–60.7)	53.0 (48.6–60.0)
17-OHProgesterone (ng/mL) (median (Q1–Q3))	2.4 (1.7–3.6)	2.1 (1.7–10.3)	2.6 (1.9–4.2)	2.4 (1.9–4.0)	2.2 (1.5–3.0)	44.4 (2.1–5.9)	1.6 (1.2–2.3)
TSH (µIU/mL) (median (Q1–Q3))	2.1 (1.5–2.8)	2.3 (1.0–4.4)	2.0 (1.6–2.4)	2.0 (1.5–2.8)	2.1 (1.4–2.9)	2.0 (1.5–3.1)	1.9 (1.3–3.4)

## Data Availability

The original contributions presented in this study are included in the article. Further inquiries can be directed to the corresponding author.

## Abbreviations

The following abbreviations are used in this manuscript:

25(OH)D	25-hydroxivitamn D
BMI	Body Mass Index
DHEAS	Dehydroepiandrosterone-sulphate
FSH	Follicle-stimulating Hormone
IR	Insulin Resistance
HDL	High-density lipoprotein
HOMA	Homeostatic model assessment
LH	Luteinizing hormone
OGTT	Oral glucose tolerance test
PCOS	Polycystic Ovary Syndrome
SHBG	Sex hormone-binding globulin
TSH	Thyroid-stimulating hormone
